# Comparison of retinal nerve fiber layer thickness between normal population and patients with diabetes mellitus using optical coherence tomography

**DOI:** 10.12669/pjms.35.1.65

**Published:** 2019

**Authors:** Mohammad Asim Mehboob, Zulfiqar Ali Amin, Qamar Ul Islam

**Affiliations:** 1 Dr. Mohammad Asim Mehboob, FCPS (Ophth), FICO, MRCSEd (Ophth), Combined Military Hospital (CMH) Gujranwala, Gujranwala, Pakistan; 2 Dr. Zulfiqar Ali Amin, FCPS(Med), FCPS (Oncology), Associate Professor, PNS Shifa Naval Hospital, Karachi, Pakistan; 3 Dr. Qamar Ul Islam, FCPS (Ophthalmology), FCPS (Vitreo-retinal Ophthalmology), Associate Professor, PNS Shifa Naval Hospital, Karachi, Pakistan

**Keywords:** Diabetic neuropathy, Optical coherence tomography, Retinal nerve fibre layer thickness

## Abstract

**Objective:**

To compare the difference in peripapillary Retinal Nerve Fibre Layer (RNFL) thickness between normal population and Type-II diabetic patients without diabetic retinopathy using Spectral Domain Optical Coherence Tomography (SD OCT).

**Methods:**

This cross sectional study was carried out at PNS Shifa Naval Hospital, from May 2017 to November 2017. Out of 200 eyes, 100 eyes were of normal individuals and 100 eyes were of Type-II diabetic patients without diabetic retinopathy. Both groups were age and gender matched. Average RNFL thickness, along with RNFL of each quadrant of individuals was noted using SD OCT, and compared between two groups.

**Results:**

Mean age of study population was 44.63 ± 4.30 years. Mean axial length was 23.46 ± 0.59 mm. Mean peripapillary RNFL thickness was 126.98 ± 10.07 µm in Group-A (normal individuals), and 120.77 ± 5.41 µm in Group-B (Type-II diabetics). Difference in mean RNFL thickness, as well as RNFL thicknesses of each quadrant was statistically significant between both groups (p-value < 0.001).

**Conclusion:**

Diabetic patients have thin RNFL as compared to normal individuals, and must be taken in account while making diagnosis of any disease based on thinning of RNFL.

## INTRODUCTION

Diabetes Mellitus (DM) is a commonly encountered micro vascular disorder in ophthalmic outpatient department. Its epidemiology shows a rapid increase in number of diagnosed and undiagnosed cases, and it is estimated that approximately 191 million people may be suffering from this disorder by year 2030. The same study also estimates that people with severely impaired eyesight may also increase to approximately 56.3 million, if prompt actions at social and policy making level are not taken.^1^ In a resources limited country like Pakistan, the figure is expected to cross 14 million people by year 2040. The country is expected to be ranked 8th in the country, by year 2040, in terms of number of people suffering from DM.^2^ The condition affects major vital organ and organ systems of the body, and is frequently accompanied with neuropathy, nephropathy, cardiovascular disorders and retinopathy. Diabetic neuropathy has a profound effect on quality of life of diabetic patients. Research is ongoing for early detection of diabetic neuropathy, and finding pharmaceutical cure of this disabling condition. Assessment of Retinal Nerve Fiber Layer (RNFL) thickness has been in clinical practice for early detection of glaucoma.^3^ Since glaucoma is a form of optic neuropathy, researchers have found variation in RNFL thickness in patients suffering from various neuropathies like migraine, Alzheimer’s disease, multiple sclerosis, obstructive sleep apnea and neurofibromatosis.^4^-^8^

Various studies have shown the difference in RNFL thickness in diabetic patients, as compared to normal age matched population.^9^ Diabetic neuropathy is supposed to affect neurons, and thus, measurement of RNFL thickness in patients with DM can be utilized to diagnose patients with, or at risk for development of diabetic neuropathy. The aim of study was to compare the difference in peripapillary Retinal Nerve Fibre Layer (RNFL) thickness between normal population and Type-II diabetic patients without diabetic retinopathy using Spectral Domain Optical Coherence Tomography (SD OCT).

## METHODS

This cross sectional study was carried out at PNS Shifa Naval Hospital, from May 2017 to November 2017, after approval from the institutional ethical review committee, and taking written informed consents from patients. A total of 200 eyes of 200 patients were analyzed. Patients from either gender, aged 40-60 years, either normal individuals, or with diagnosis of DM on basis of fasting blood sugar levels, and HBA1C, but no fundoscopic evidence of diabetic retinopathy were included. Patients with glaucoma, diabetic retinopathy, high axial myopia, tilted disc, family history of glaucoma, hereditary optic neuropathies, ocular trauma, ocular surgery, chronic topical steroids users, hypermetropia, and previous laser photocoagulation were excluded. Demographic data of study population was acquired. All patients underwent detailed ophthalmic examination with measurement of best corrected visual acuity, anterior and posterior segment examination, measurement of intraocular pressure, fundus examination with non-contact fundus viewing lens. All examination was done by single vitreo-retinal surgeon to exclude bias. Patients were divided in two groups. Group-A was control group, with normal individuals. Group-B was diagnosed Type-II DM patients, with good glycemic control, but without evidence of diabetic retinopathy. Axial length measurement was performed using partial laser interferometry (IOL Master, Carl Zeiss Meditec, Dublin, CA, USA).

Average of three readings was recorded for data analysis. Peripapillary RNFL measurements were taken using Spectralis SD OCT system (Heidelberg Engineering GmbH, Heidelberg, Germany) after dilating one eye with 0.1% topical Tropicamide eye drops. Global, as well as mean peripapillary RNFL thickness of all four quadrants was taken. The pre devised proforma was completed by researcher endorsing subject’s demography, ocular examination findings and RNFL thickness of peripapillary area. Confidentiality of the patient’s record was maintained.

Statistical Package for Social Sciences (SPSS 20.0) for windows was used for statistical analysis. Descriptive statistics i.e. mean ± standard deviation for quantitative values (age, axial length, RNFL thickness) and frequencies along with percentages for qualitative variables (gender, laterality of eyes) were used to describe the data. Shapiro Wilk’s test was used to check normality of data. After normality testing, qualitative variables were compared between two groups using Chi Square test and quantitative variables were compared using independent ‘t’-test. A p-value of ≤0.005 was considered statistically significant.

## RESULTS

Two hundred eyes of 200 subjects fulfilling the inclusion criteria were analyzed. Mean age of study population was 44.63 ± 4.30 years (Range: 40-50 years). 115 (57.5%) subjects were males, while 85 (42.5%) were females. Mean axial length was 23.46 ± 0.59 mm (Range: 22.2-24.5 mm). Demographic data of study population and of both groups is given in Table-I. There was no statistically significant difference in both groups in terms of age, gender, laterality of eyes and axial length (p-value =0.623, 0.866, 0.258 and 0.759 respectively). Mean of peripapillary RNFL thickness, along with mean peripapillary RNFL thickness of superior, inferior, nasal and temporal quadrants of study population and both groups is given in Table-II. The difference in mean RNFL thickness, as well as RNFL thickness of all four quadrants was statistically significant between both groups (p<0.001).

**Table-I:** Group wise demographic data (n=200).

Characteristic	Study Population (n=200)	Group-A (n=100)	Group-B (n=100)	p-Value
Age (Years) Mean ± SD	44.63 ± 4.30	44.48 ± 4.34	44.78 ± 4.28	0.623*
*Gender*				
Male	115 (57.5%)	57(57%)	58(58%)	0.886**
Female	85 (42.5%)	43(43%)	42(42%)
*Eye*				
Right	104(52%)	48(48%)	56(56%)	0.258**
Left	96(48%)	52(52%)	44(44%)	
Axial Length (mm) Mean ± SD	23.46 ± 0.59	23.45 ± 0.58	23.47 ± 0.61	0.759*

* Independent t-test,

** Chi Square test.

**Table-II:** Group wise distribution of peripapillary RNFL thickness (n=200).

Quadrant	Study Population (n=200)	Group-A (n=100)	Group-B (n=100)	p-Value*
Average (µm) Mean ± SD	123.87 ± 8.64	126.98 ± 10.07	120.77 ± 5.41	< 0.001
Superior (µm) Mean ± SD	154.36 ± 8.33	158.74 ± 8.35	149.97 ± 5.57	< 0.001
Inferior(µm) Mean ± SD	146.06 ± 12.80	151.86 ± 14.36	140.26 ± 7.42	< 0.001
Nasal (µm) Mean ± SD	102.89 ± 4.42	104.34 ± 4.04	101.44 ± 4.31	< 0.001
Temporal (µm) Mean ± SD	97.56 ± 10.63	103.70 ± 9.09	91.42 ± 8.27	< 0.001

* Independent t-test.

## DISCUSSION

We measured RNFL thickness in diabetic patients and controls, keeping them age matched (40-60 years) and excluding the extreme of axial lengths. RNFL measurement is affected by various factors, most significant being age, gender and axial length.^10^ There was no difference among two groups in terms of age, gender, laterality of eyes and axial length (p=0.623, 0.886, 0.258 and 0.759 respectively). Thus the two groups were matched in terms of these variables, and the difference in RNFL measurement can be attributed to neurodegenerative effect of DM alone. In a study by Altman C and associates, it was highlighted that diabetic retinopathy is a combination of micro vascular abnormalities combined with neurodegenerative effect on retinal ganglion cells. They also pointed out that neurodegenerative effect of DM may precede the micro vascular abnormalities.^11^ The same was also advocated by another study, pointing out that diabetic neuro degenerative effect on ganglion cells and RNFL thickness is primary due to DM, and secondly as after effect of damage of blood retinal barrier and resultant damage to retinal ganglions due to edema and increase in extracellular fluid levels.^12^

We compared RNFL thickness of controls with DM patients without retinopathy. The aim was two folds. One was to evaluate, whether neurodegenerative effect of DM precedes the development of diabetic retinopathy. Secondly, it was assumed that diabetic retinopathy affects different measurements of retinal areas by SD OCT, and results may get affected due to change in thickness due to edema, hemorrhages and cotton wool spots. In a similar study conducted by Sohn EH and associates, it was revealed that patients with DM but no diabetic retinopathy had thin ganglion cell layer and RNFL, as compared to age matched control group. They also found out that the thinning was observed over a period of four years, and was independent of age, gender and glycosylated haemoglobin levels.^13^ Thus, we have evidence to assume that the retinal neuro degeneration may precede the development of diabetic retinopathy. In another study conducted on Type-I diabetic patients, without retinopathy, similar difference in RNFL thickness was found.^14^ In another study conducted on Type-I diabetic children, without retinopathy, it was found that RNFL and ganglion cell thickness was far less as compared to normal children.^15^ Thus there is evidence of neuro generative effect of DM on ganglion cells before the development of vascular component of diabetic retinopathy.

Few studies have found different results. In a study conducted on Type-II diabetic patients with diabetic retinopathy, it was observed that the RNFL was thinner in superonasal and superotemporal quadrant only, as compared to normal individuals. They advocate that neuro degeneration is an early component of DM, and may occur alongside diabetic retinopathy, but the preferential thinning of two quadrants only still needs to be explained.^16^ In a similar study conducted on Type-1 diabetic patients without retinopathy, RNFL thickness was measured with help of scanning laser polarimetry and it was observed that the superior quadrant showed thinning, as compared to normative data base.^17^ These two studies have shown that irrespective of presence of retinopathy, the superior quadrants show RNFL thinning in Type-1 diabetic patients, irrespective of age, and the neurodegeneration may precede the development of diabetic retinopathy vascular component.

Our findings are different than research conducted by Srinivasan S and co-workers, who found that both RNFL thickness and ganglion cell layer thickness in healthy individuals, and diabetics with or without retinopathy was not statistically different in any quadrant.^18^ In another such study to examine the neuronal structural integrity of cornea and retina as markers for neuronal degeneration in diabetic individuals, it was found that RNFL thickness was not different amongst groups with or without retinopathy, and amongst control group.^19^

We did not compare the RNFL thickness between diabetic patients with and without retinopathy. In a study conducted to compare RNFL thickness between normal individuals, diabetics with and without retinopathy, it was observed that RNFL was characteristically thinner as the retinopathy progressed.^20^ Thus, it is assumed that vascular progression of diabetic retinopathy is accompanied by similar progression in neurodegeneration of ganglion cells, causing further thinning of RNFL. In another study, it was observed that duration of diabetes had an inverse relation with RNFL thickness, and with progression in retinopathy and duration of diabetes, the RNFL got thin.^21^

### Limitation of study

Our study has limitations of not including the patients with diabetic retinopathy, and including only diabetic patients with controlled blood sugar levels. Inclusion of patients with retinopathy and uncontrolled blood sugar profile, and measurement of ganglion cell layer thickness will yield more reproducible results with better clinical implications.

## CONCLUSION

We conclude that RNFL thickness is reduced in patients with DM, as compared to age matched controls. The thinning of RNFL thickness is indirect evidence of neurodegeneration due to DM, which may precede the development of diabetic retinopathy, and is irrespective of age, glycemic control or duration of diabetes.
